# Mode of Delivery and Offspring Body Mass Index, Overweight and Obesity in Adult Life: A Systematic Review and Meta-Analysis

**DOI:** 10.1371/journal.pone.0087896

**Published:** 2014-02-26

**Authors:** Karthik Darmasseelane, Matthew J. Hyde, Shalini Santhakumaran, Chris Gale, Neena Modi

**Affiliations:** Section of Neonatal Medicine, Department of Medicine, Imperial College London, Chelsea & Westminster Hospital campus, London, United Kingdom; Yale School of Public Health, United States of America

## Abstract

**Background:**

It has been suggested that mode of delivery, a potentially powerful influence upon long-term health, may affect later life body mass index (BMI). We conducted a systematic review and meta-analysis of the effect of Caesarean section (CS) and vaginal delivery (VD) on offspring BMI, overweight (BMI>25) and obesity (BMI>30) in adulthood. Secondary outcomes were subgroup analyses by gender and type of CS (in-labour/emergency, pre-labour/elective).

**Methods:**

Using a predefined search strategy, Pubmed, Google Scholar and Web of Science were searched for any article published before 31^st^ March 2012, along with references of any studies deemed relevant. Studies were selected if they reported birth characteristics and long-term offspring follow-up into adulthood. Aggregate data from relevant studies were extracted onto a pre-piloted data table. A random-effects meta-analysis was carried out in RevMan5. Results are illustrated using forest plots and funnel plots, and presented as mean differences or odds ratios (OR) and 95% confidence intervals.

**Results:**

Thirty-five studies were identified through the search, and 15 studies with a combined population of 163,753 were suitable for inclusion in the meta-analysis. Comparing all CS to VD in pooled-gender unadjusted analyses, mean BMI difference was 0·44 kg·m^-2^ (0·17, 0·72; *p* = 0·002), OR for incidence of overweight was 1·26 (1·16, 1·38; *p*<0·00001) and OR for incidence of obesity was 1·22 (1·05, 1·42; *p = *0·01). Heterogeneity was low in all primary analyses. Similar results were found in gender-specific subgroup analyses. Subgroup analyses comparing type of CS to VD showed no significant impact on any outcome.

**Conclusions:**

There is a strong association between CS and increased offspring BMI, overweight and obesity in adulthood. Given the rising CS rate worldwide there is a need to determine whether this is causal, or reflective of confounding influences.

**Systematic review registration:**

An *a priori* protocol was registered on PROSPERO (registration number: CRD42011001851)

## Introduction

The last twenty years have seen worldwide increases in obesity prevalence in children and adults, with the highest incidences reported in the USA and Scotland (33·8% and 30% respectively) [1]. In England, adult obesity has risen from 16·4% to 26% between 1995 and 2010 [1], [2] at an estimated cost to the National Health Service of >£5·1billion annually [3].

Concurrently, between 1990 and 2008, there has been a 100% increase in Caesarean Section (CS) births in England [4]. The World Health Organisation (WHO) recommends that the CS rate should not exceed 15% [5]. However many countries report higher rates; including China (60%) [6], Brazil (47%) [7], and England (23·8%) [8].

Adverse effects of CS on the neonate immediately post-partum are widely recognised. CS is associated with the highest rates of neonatal morbidity and mortality of all modes of delivery [9], with increased risk of a low 1-minute Apgar [10], respiratory distress, hypoglycaemia and a prolonged stay in a neonatal intensive care unit [11].

Controversially, it has been suggested that birth by CS predispose offspring to adverse health outcomes in childhood [9]. A 20% increased odds of asthma and type-1 diabetes, and a 23–32% increased odds of atopic disorders have been reported. An association between CS and later-life obesity has also been postulated. A study conducted in North America, evaluating almost 200,000 adolescents, reported that children born by CS are 40% more likely to be overweight [12]. To date studies addressing the possibility of an association between CS and adult obesity have been small and contradictory. Goldani *et* al [13] in a Brazilian cohort study of young adults aged 23–24, demonstrated that the odds of obesity are increased by 50% following birth by CS. Conversely another Brazilian study evaluating obesity in 4297 adults showed no difference by mode of delivery [14]. A recent meta analysis published by Li *et al* [64] presented adult data from 3 studies (n = 6,807) that specifically examined the effect of mode of delivery and offspring overweight/obesity, demonstrating an adjusted pooled OR of 1.50 (95%CI 1.02, 2.20, I^2^ = 74%).

We aimed to perform a systematic review and meta-analysis to identify any association between mode of delivery and offspring body mass index (BMI), and risk of overweight, and obesity in adulthood. We also sought to determine if offspring age, gender and type of CS had an effect on outcomes. In contrast to Li *et al* [64] we aimed to include all cohorts reporting data on adult BMI and weight, together with mode of delivery, independent of whether or not the association between the two was studied or published.

## Methods

A systematic review of studies reporting adult anthropometry (BMI, height, weight, incidence of overweight/obesity) by mode of delivery was conducted using an *a priori* protocol (registered on PROSPERO [15]) following PRISMA guidelines for reporting systematic reviews and meta-analyses [16].

### Definition of exposure and outcomes

Outcomes studied were offspring BMI, overweight and obesity in adulthood (≥18 years). Overweight and obesity were classified according to the National Institute of Clinical Excellence categories [17], namely “obese” (BMI>30) and “overweight or obese” (BMI>25, including BMI>30). Type of exposure was classed as vaginal delivery (VD) (including natural, forceps and vacuum extraction) and CS, with CS groups further categorised as Pre-Labour CS (pre-labour-CS) or In-Labour CS (in-labour-CS).

### Literature search

A search was conducted in PubMed (http://www.ncbi.nlm.nih.gov/) for any studies published before 31^st^ March 2012, using the following search strategy using PubMed Medical Subject Heading (MeSH) terms: *“(Parturition OR Delivery, Obstetric OR Cesarean) AND (obesity OR Body Mass Index OR Overweight)”*, limited to human studies. A similar search of Google Scholar (http://scholar.google.co.uk/) and Web of Knowledge (http://apps.webofknowledge.com *)* was also carried out using the search strategy “*(caesarean OR cesarean OR caesarian OR cesarian) AND (obesity OR Body Mass Index OR Overweight) AND (Adult) AND (Offspring)”*. No limits on language, country, study type or publication period were applied. Foreign language publications were translated using “Google Translate” (http://translate.google.com/).

### Study selection

Titles and abstracts of identified studies were independently screened by two reviewers [KD and MJH]. If abstracts were unavailable, the full text was obtained. The full texts of relevant abstracts were appraised for inclusion [KD and CG]. Any disagreement over eligibility of a study was referred to all authors. For inclusion a study must have reported either: (1) both mode of delivery and adult offspring BMI; (2) mode of delivery with long-term offspring follow-up (into adulthood) or (3) adult offspring BMI with birth characteristics. References of included studies were hand searched for relevant publications. Review articles and letters to editors were excluded after reference lists were searched. If multiple papers reported data from the same cohort, the study reporting BMI at an age closest to the median age across all studies was included.

### Data extraction

A basic dataset was extracted from each study using a pre-piloted data collection form [KD and SS]. In studies where required data were not reported or only adjusted results provided, authors were contacted to request these [KD and MJH]. If no response was received after two emails or relevant data were unavailable, the study was excluded from the meta-analysis. Where authors were not contactable, the principal investigator of the cohort was approached. Where mean and SD for BMI was provided by gender only, combined means and SD for both genders together were calculated.

Methodological quality of each study was assessed using a modified Newcastle-Ottawa Scale [18] [KD and SS]; studies are scored based on population selection, study comparability and outcome (Figure S1).

### Data analysis

Meta-analyses were carried out using the inverse-variance method for mean BMI difference and the Mantel-Haenszel method for overweight and obesity odds ratios (OR) in RevMan 5 (5.0.23) to identify any association between mode of delivery and adult BMI, overweight and obesity separately. Forest plots were created using RevMan 5 (5.0.24). Funnel plots were created and Egger's Test [22] performed in Stata 12 to investigate publication bias and other small-study effects. Differences between groups are provided as pooled estimated mean differences with 95% confidence interval (CI) or unadjusted OR with 95% CI.

### Between study heterogeneity

Random-effects models were used throughout as it was considered unlikely that the effect of interest was the same across all studies, invalidating the main assumption for fixed-effect models. Heterogeneity (between study variation) was assessed using the chi-squared test for Cochrane's Q statistic and by calculating I^2^, the estimated proportion of variance in the study outcome due to heterogeneity [19], [20].

However, where heterogeneity was low (p>0·05 from the chi-squared test and I^2^<50%), a fixed-effects model was carried out to check the sensitivity of findings as a random effects analysis can give greater weight to smaller studies. As heterogeneity tests have low power when study numbers are small [21], a fixed-effects meta-analysis was not carried out for analyses with less than five studies. If I^2^ was 0% a fixed-effects analysis was also not carried out as results would be identical to the random-effects model.

For all random effects analyses 95% prediction intervals (PI) were calculated as this provides a useful measure of the effects we would expect to see in future studies [23]. The prediction interval is the range in which 95% of individual study effects are expected to lie, in contrast to the confidence interval which is for the average effect across all studies. The limits are given by 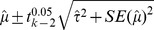 where 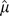 is the pooled estimate of the average effect, 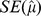 its standard error, and 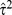 the estimate of between study variance. A t distribution on k-2 degrees of freedom (where k is the number of studies) is used rather than a normal distribution because 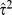 is estimated with uncertainty.

Pre-specified subgroup analyses were carried out by gender and type of CS (in-labour-CS/pre-labour-CS) for studies providing relevant data. Where a substantial difference in the magnitude of BMI difference was shown between subgroups, statistical significance was tested using meta-regression. Meta-regression was also used to determine whether study results varied with age of offspring.

If type of CS data were available for only a subgroup of studies, to confirm that any difference in results was not due to a subgroup effect, the mean BMI difference, overweight OR and obesity OR was calculated for the subgroup comparing all CS deliveries (i.e. not separated into pre-labour-CS or in-labour-CS) to VD.

## Results

### Search results

**Figure 1:**
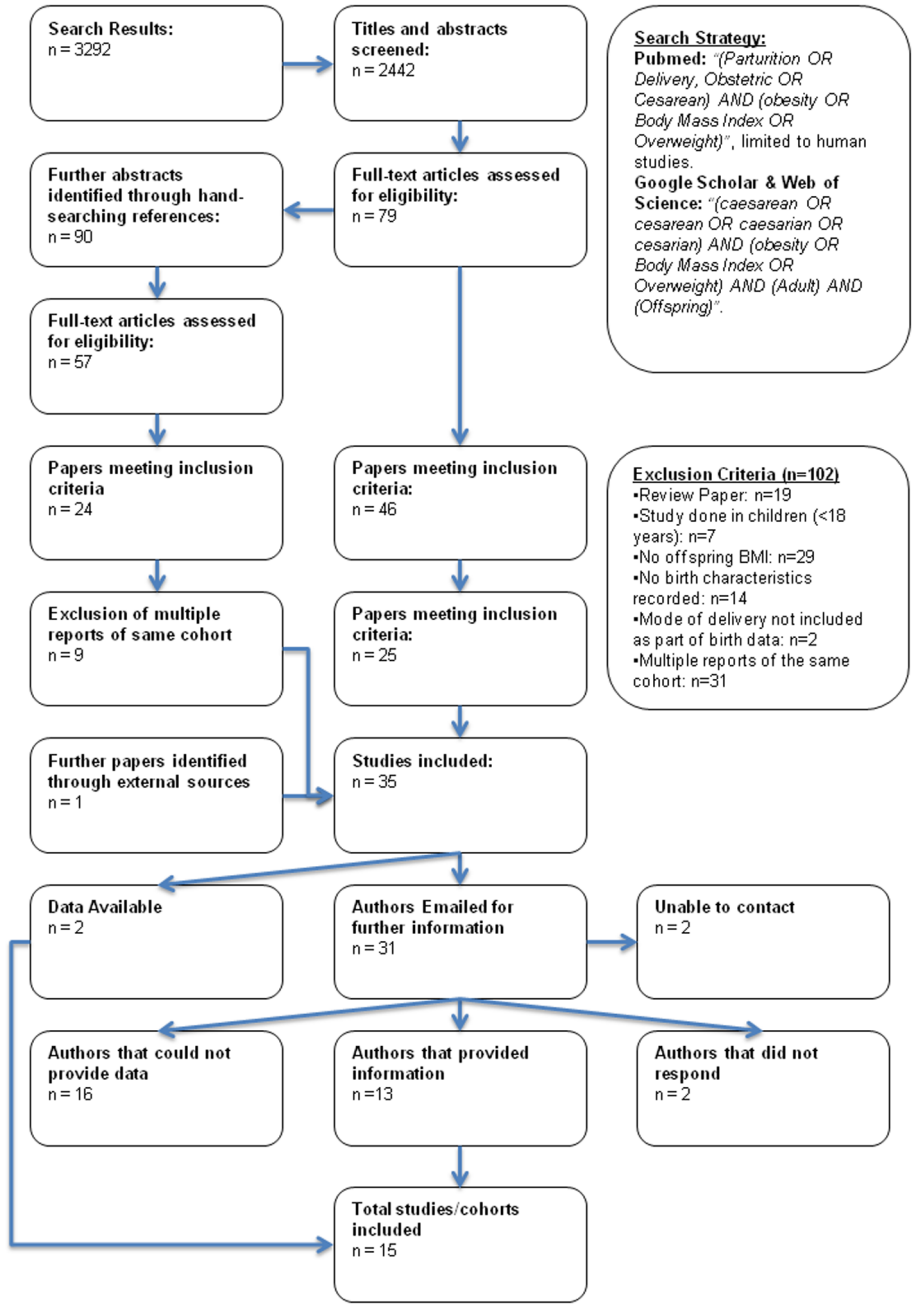
Flow chart of the search strategy used in the review. The relevant number of papers at each point is given.

The search yielded 3292 abstracts (PubMed: 1413; Google Scholar: 1850; Web of Science: 29). Google Scholar imposes a 1000 result limit, so only the first 1000 abstracts were screened. Consequently, 2442 titles and abstracts were screened for inclusion. Seventy-nine abstracts appeared relevant; through hand-searching their references a further 90 abstracts were screened of which 57 appeared relevant. A further paper was identified through external means. Duplicate datasets, and papers which on full examination evidently had not collected mode of delivery data, were removed, leaving 35 studies with apparently relevant data (**Figure 1**).

The authors of 30 studies were contacted [13], [14], [24]–[51]; one cohort was contacted directly [52]; two cohort studies were open-access [53]–[60]; and in two cases we were unable to contact the authors [61], [62]. Twenty-nine authors replied [13], [14], [24]–[47], [50]–[52] of whom 13 provided additional data suitable for inclusion [13], [14], [39]–[47], [50], [51].

The 1958 British Birth Cohort dataset included implausible BMI values, so limits were imposed (excluding subjects with BMI>200 kg·m^−2^ and BMI<10 kg·m^−2^). Mean (SD) BMI was calculated following exclusion of these implausible data.

The 1924 Helsinki Birth Cohort [44] had a small CS group (n = 5), so was only included in the overall meta-analyses. The single gender studies of Cnattingius *et al* [47], (female); the GOOD Study [41], (male); and Svensson *et al* [51], (male) were excluded from pooled gender analyses. One study [46] did not provide data on incidence of obesity, and was therefore excluded from the obesity analyses.

In total, 15 studies were included (combined population 163,753). Study descriptions and data are shown in **table 1**.

**Table 1:** Studies included in this meta-analysis examining the association between mode of delivery and offspring BMI in adulthood.

Authors	Study Details	Factors adjusted for	NOS	Follow-up Rate	Population Size	Mean Age	Sex	MoD	Mean + SD BMI	N	% of Sample BMI > 25 (n)	% of Sample BMI > 30 (n)	
**1934 Helsinki Birth Cohort^40^**	PC; Finland; Persons born in Helsinki during 1934–1944 at University Central Hospital that still lived in Finland in 1971. All living members were sent a questionnaire in 2000, and a random sample of 2901 was invited for clinical examination. Of this sample data were obtained on 1999. Investigators were blinded to birth data.	N/A	5*	68.9	1999	61.6	**M**	**VD**	27.5 (4.3)	899	73.53 (661)	22.25 (200)	
								**CS**	27.7 (2.8)	28	85.71 (24)	25 (7)	
							**F**	**VD**	27.7 (5)	1056	67.71 (715)	27.46 (290)	
								**CS**	27.7 (5.7)	18	66.67 (12)	16.67 (3)	
							**Total**	**VD**	27.6 (4.7)	1955	70.38 (1376)	25.06 (490)	
								**CS**	27.7 (4.1)	46	78.26 (36)	21.74 (10)	
**1958 British Birth Cohort^51^**	PC; UK; All births in England, Scotland and Wales between 3^rd^–9^th^ March 1958. Height and weight were measured during an interview when subjects were 33 for 10,490 subjects from a target sample of 15,667. Pre-terms, pregnant women and offspring from multiple pregnancies were excluded. Investigators were blinded to birth data.	N/A	5*	67	10490	33	**M**	**VD**	25.71 (4.78)	5011	51.65 (2588)	11.28 (565)	
								**CS**	26.21 (4.17)	145	55.17 (80)	14.48 (21)	
							**F**	**VD**	24.66 (5.56)	5209	35.71 (1860)	12.06 (628)	
								**CS**	25.61 (5.70)	125	40.8 (51)	16.8 (21)	
							**Total**	**VD**	25.18 (5.218)	10220	43.52 (4448)	11.67 (1193)	
								**CS**	25.93 (4.937)	270	48.52 (131)	15.56 (42)	
								**IL-CS**	26.24 (5.278)	154	49.35 (76)	18.18 (28)	
								**PL-CS**	25.53 (4.435)	116	47.41 (55)	12.06 (14)	
**1970 British Birth Cohort^59^**	PC; UK; All children born in Great Britain between 5^th^ – 11^th^ April 1970, of which 9316 were followed up at 34 years old. Height and weight data at 34 was obtained by confidential self-reporting. Investigators were blinded to birth data	N/A	4*	52.12	8634	34	**M**	**VD**	26.661 (4.579)	3983	61.11 (2434)		17.9 (712)
								**CS**	26.624 (4.677)	189	59.26 (112)		19.6 (37)
							**F**	**VD**	25.170 (5.160)	4267	40.47 (1727)		15.4 (658)
								**CS**	25.705 (5.206)	195	46.67 (91)		15.9 (31)
							**Total**	**VD**	25.89 (4.944)	8250	50.44 (4161)		16.61 (1370)
								**CS**	26.16 (4.968)	384	52.86 (203)		17.71 (68)
**ACONF^39^**	PC; Scotland; Survey of all primary school children aged 6-12 in Aberdeen 1962-1964. Follow-up information in 2001-2003 was conducted via postal questionnaire when participants were aged 43-53. Investigators were blinded to birth data.	N/A	3*	61.6	6948	43–53	**M**	**VD**	26.83 (4.043)	3233	64.06 (2071)		18.65 (603)
								**CS**	26.877 (3.854)	103	67.96 (70)		19.42 (20)
							**F**	**VD**	26.21 (5.444)	3505	49.61 (1739)		19.06 (668)
								**CS**	26.398 (5.507)	107	53.27 (57)		20.56 (22)
							**Total**	**VD**	26.51 (4.832)	6738	56.54 (3810)		18.86 (1271)
								**CS**	26.63 (4.763)	210	60.48 (127)		20 (42)
								**IL-CS**	26.46 (4.777)	91	57.14 (52)		17.58 (16)
								**PL-CS**	26.76 (4.769)	119	63.03 (75)		21.85 (26)
**Barros ** ***et al*** **, 2012^14^**	PC; Brazil; All live babies born in 1982 in Pelotas were recruited and then traced and in 2004–2005; blinding not stated.	Sex; birthweight; physical activity; schooling at time of assessment; family income at time of assessment; smoking; alcohol consumption; family income at birth; method of payment for the delivery and maternal factors: skin colour; age; parity; smoking; height; pre-pregnancy weight.	5*	75.34	4288	22.8	**M**	**VD**	23.6 (4.08)	1583	27.61 (437)		7.3 (116)
								**CS**	24.3 (4.01)	623	38.2 (238)		8.2 (51)
							**F**	**VD**	23.5 (4.66)	1503	26.75 (402)		8.9 (134)
								**CS**	23.7 (4.63)	579	27.63 (160)		9.7 (56)
							**Total**	**VD**	23.551 (4.376)	3086	27.19 (839)		8.1 (250)
								**CS**	24.011 (4.328)	1202	33.11 (398)		8.9 (107)
**Goldani ** ***et al*** **, 2011^13^**	PC; Brazil; A random sample of singleton-born subjects from a cohort of all births registered in Ribeirão Preto between 1^st^ June 1978 to 31^st^ May 1979 that were still alive and living in the city between 2002–2004; all investigators and personnel were blinded to birth data. Of note there were some differences described between the sample and the whole cohort.	Sex; SES; birthweight; smoking, education; physical activity; maternal factors: smoking; education;	7*	97.81	2057	23.9±0.71	**M**	**VD**	24.6 (4.23)	675	39.85 (269)		10.67 (72)
								**CS**	25.9 (4.76)	317	50.47 (160)		17.35 (55)
							**F**	**VD**	23.3 (4.78)	725	27.45 (199)		10.21 (74)
								**CS**	24.2 (5.59)	340	32.35 (110)		13.24 (45)
							**Total**	**VD**	23.927 (4.568)	1400	33.43 (468)		10.43 (146)
								**CS**	25.02 (5.271)	657	41.1 (270)		15.22 (100)
**Helsinki Study of VLBWA^46^**	PC; Finland; Control group from the VLBWA cohort; subjects recruited were the next available singleton infant born at term of the same sex who was not small for gestational age; investigators blinded to birth data.	N/A	5*	54.8	176	22.49	**M**	**VD**	23.37 (3.307)	60	31.67 (19)		-
								**CS**	22.61 (2.597)	12	8.33 (1)		-
							**F**	**VD**	22.55 (3.723)	96	19.79 (19)		-
								**CS**	25.7 (1.902)	8	50 (4)		-
							**Total**	**VD**	22.87 (3.58)	156	24.36 (38)		-
								**CS**	23.85 (2.768)	20	25 (5)		-
								**IL-CS**	23.18 (2.319)	11	27.27 (3)		-
								**PL-CS**	24.655 (3.181)	9	22.22 (2)		-
**Kajantie ** ***et al*** **, 2003^44^**	PC; Finland; Persons born between 1924 and 1933 at the Helsinki University Central Hospital who resided in Finland in 1971, and remained in Finland thereafter. Investigators were blinded to birth data.	N/A	4*	7.1	500	69.6	**Total**	**VD**	27.61 (4.358)	495	71.72 (355)		24.44 (121)
								**CS**	26.44 (2.906)	5	60 (3)		20 (1)
**Mi ** ***et al*** **, 2000^42^**	PC; China; Adult offspring of women who had sequential, live, singleton births at Peking Union Medical College between July 1948 to December 1954, still residing in Beijing. Height and weight data were measured by one of two observers. Investigators were blinded to birth data.	N/A	4*	86.6	628	45	**M**	**VD**	24.74 (3.07)	256	38.67 (99)		5.86 (15)
								**CS**	24.25 (3.23)	53	45.3 (24)		3.77 (2)
							**F**	**VD**	23.31 (3.07)	266	24.81 (66)		2.26 (6)
								**CS**	23.58 (3.25)	53	26.42 (14)		3.77 (2)
							**Total**	**VD**	24.01 (3.149)	522	31.61 (165)		4.02 (21)
								**CS**	23.915 (3.242)	106	35.85 (38)		3.77 (4)
**Mysore Cohort^45^**	PC; India; All singletons born alive at Holdsworth Memorial Hospital, Mysore between 1934 to 1954, living within 8 square miles of the hospital, identified between 1993 to 2004. Investigators were blinded to birth data.	N/A	4*	4.3	1064	46.8	**M**	**VD**	23.09 (4.201)	536	33.02 (177)		5.04 (27)
								**CS**	24.2 (4.3)	14	35.71 (5)		7.14 (1)
							**F**	**VD**	25.546 (5.267)	507	53.85 (273)		16.96 (86)
								**CS**	25.6 (4.4)	7	57.14 (4)		14.29 (1)
							**Total**	**VD**	24.263 (4.932)	1043	43.15 (450)		10.83 (113)
								**CS**	24.6 (4.3)	21	42.857 (9)		9.52 (2)
**Vonk ** ***et al*** **, 2004^43^**	PC; Netherlands; All newborn babies in the Department of Obstetrics of the University Hospital in Groningen between 1975 to 1978, were sent a postal questionnaire in 1998. All responders to the questionnaires were invited for medical characterisation where height and weight were measured.	N/A	4*	38	590	20.49	**M**	**VD**	22.5 (2.7)	248	14.9 (37)		1.2 (3)
								**CS**	22.9 (3.4)	17	23.5 (4)		5.9 (1)
							**F**	**VD**	23.4 (4.0)	300	26.7 (80)		7.7 (23)
								**CS**	23.1 (2.9)	25	28 (7)		0 (0)
							**Total**	**VD**	23.0 (3.5)	548	21.4 (117)		4.7 (26)
								**CS**	23.0 (3.1)	42	26.2 (11)		2.4 (1)
								**IL-CS**	23.2 (3.2)	25	32 (8)		4 (1)
								**PL-CS**	22.7 (3.0)	17	17.6 (3)		0 (0)
**GOOD Study^41^**	RC; Sweden; study subjects were randomly identified through national population registers. Inclusion criteria were male subjects aged 18–20, willing to participate in the study. Detailed birth records were identified for these subjects. Height and weight readings were taken by trained personnel. Investigators were blinded to birth data.	N/A	3*	94.3	1008	18.9±0.6	**M**	**VD**	22.351 (3.278)	867	16.15 (140)		3.91 (34)
								**CS**	22.624 (3.146)	141	14.89 (21)		2.13 (3)
								**IL-CS**	22.505 (3.444)	70	15.7 (11)		1.43 (1)
								**PL-CS**	22.741 (2.842)	71	14.1 (10)		2.82 (2)
**Cnattingius ** ***et al*** **, 2011^47^**	Record Linkage Study; Sweden; All women who were singleton-born infants (between 1973 and 1992) AND mothers of first-born singleton infants (between 1990 and 2006). Preterms and subjects with missing data were excluded. Linked with Swedish Birth Registry to find birth characteristics. Investigators were blinded to birth data.	N/A	4*	64	103941	30.3	**F**	**VD**	24.5 (4.6)	87285	35.85 (31293)		11.56 (10087)
								**CS**	25.7 (5.202)	16656	45.63 (7601)		17.84 (2972)
								**IL-CS**	25.9 (5.3)	8148	46.76 (3810)		18.65 (1520)
								**PL-CS**	25.5 (5.1)	8508	44.56 (3791)		17.07 (1452)
**Rooney ** ***et al*** **, 2011^50^**	PC; USA; A convenience sample of women with uncomplicated pregnancies were recruited in Gundersen Clinic, Ltd. and Lutheran Hospital in La Crosse, Wisconsin, from April 1, 1989 to March 30. Exclusion criteria included gestational diabetes, pre-eclampsia, cervical incompetence, or other conditions that would possibly lead to pre-term deliveries. Children from these mothers were followed up to adulthood.	N/A	4*	54	422	18–20	**M**	**VD**	24.64 (5.64)	174	30.5 (53)		15.5 (27)
								**CS**	27.93 (9.4)	11	36.4 (4)		36.4 (4)
							**F**	**VD**	23.94 (4.8)	216	29.2 (63)		11.1 (24)
								**CS**	26.93 (8.67)	21	42.9 (9)		28.6 (6)
							**Total**	**VD**	24.25 (5.2)	390	29.7 (116)		13.1 (51)
								**CS**	27.27 (8.79)	32	40.6 (13)		31.3 (10)
**Svensson ** ***et al*** **, 2013 ^51^**	Record Linkage Study; Denmark; men born as singletons between 1977 and 1983 in Northern Denmark, who presented for compulsory medical evaluation for conscription aged 18–20. Conscription records were linked to the Danish Medical Birth Registry. Investigators were blinded to birth data.	N/A	5*	100	21,051	18–20	**M**	**VD**	23.6 (3.92)	18,913	18.04 (3412)		6.37 (1205)
								**CS**	24.1 (4.22)	2138	18.52 (396)		8.98 (192)
								**IL-CS**	23.9 (4.47)	344	16.57 (57)		8.72 (30)
								**PL-CS**	23.8 (4.42)	189	18.51 (35)		8.99 (17)

**PC:** Prospective Cohort Study; **PBC:** Population-Based Cohort Study; **RC:** Retrospective Cohort Study; **NOS:** Modified Newcastle-Ottawa Scale Score; **M:** Male; **F:** Female; **VD:** Vaginal Delivery; **CS:** Caesarean Section; **IL-CS:** Emergency/In-Labour Caesarean Section; **PL-CS:** Elective/Pre-Labour Caesarean Section; **MoD:** Mode of Delivery; **BMI:** Body Mass Index; **SES:** Socio-Economic Status; **ACONF:** Aberdeen Children of the 1950s; **VLBWA:** Very Low Birth Weight Adults

### Primary analyses

**Figure 2:**
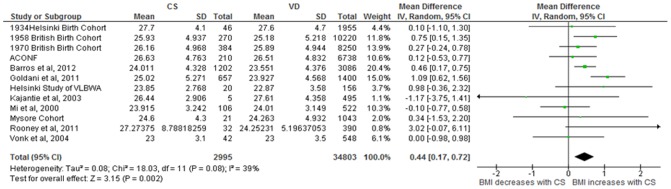
Offspring born by CS have higher BMI. Forest Plot showing the pooled gender, unadjusted mean BMI difference in adult offspring by mode of delivery.

**Figure 3:**
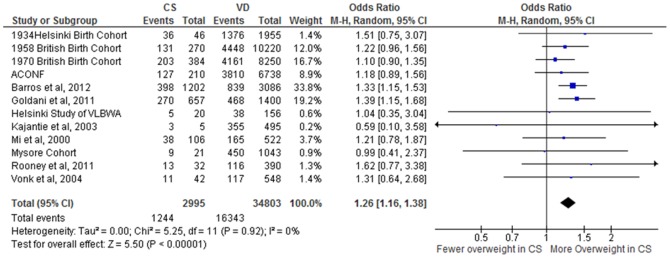
Offspring born by CS have higher incidence of overweight. Forest Plot showing the pooled gender, unadjusted OR for incidence of overweight in adult offspring, by mode of delivery.

**Figure 4:**
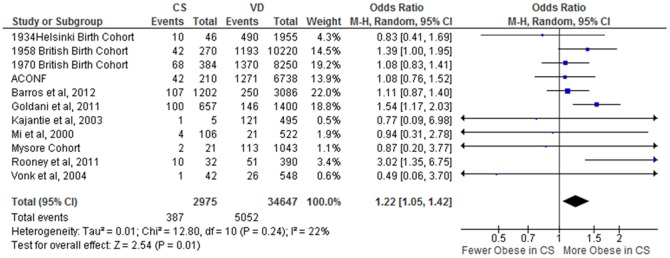
Offspring born by CS have higher incidence of obesity. Forest Plot showing the pooled gender, unadjusted OR for incidence of obesity in adult offspring by mode of delivery.

Data from 12 studies [13], [14],[39],[40],[42]–[46],[50],[53],[60] (37,798 subjects; CS = 2995, VD = 34,803) were included in the random-effects meta-analysis which showed an unadjusted mean BMI difference of 0·44 kg·m^−2^ CI [0·17, 0·72], *p* = 0·002, I^2^ = 39% (**Figure 2**). The OR for incidence of overweight following CS in comparison to VD was 1·26 CI [1·16, 1·38], *p*<0·00001, I^2^ = 0% (**Figure 3**). A population of 37,622 (CS = 2975, VD = 34,647) from 11 studies [13], [14], [39], [40], [42]–[45], [50], [53], [60] was available to investigate the association between mode of delivery and obesity; the OR for CS in comparison to VD was 1·22 CI [1·05, 1·42], *p* = 0·01, I^2^ = 22% (**Figure 4**).

### Subgroup analyses

**Men:** Data from 13 studies [13], [14], [39]–[43], [45], [46], [50], [51], [53], [60] (40,229 subjects; CS = 3791, VD = 36,438) were included. The mean difference in BMI between CS and VD was 0·42 kg·m^−2^ CI [0·18, 0·67], *p* = 0·0008, I^2^ = 40% (Figure S2A). The OR for overweight was 1·21 CI [1·02, 1·43], *p* = 0·03, I^2^ = 56% (Figure S2B). Twelve studies [13], [14], [39]–[43], [45], [50], [51], [53], [60] (40,157 subjects; CS = 3776, VD = 36,378), were suitable to investigate obesity (1·33 CI [1·16, 1·53], *p*<0·0001, I^2^ = 8% (Figure S2C)

**Women:** Data from 12 studies [13], [14], [39], [40], [42], [43], [45]–[47], [50], [53], [60] (123,069 subjects; CS = 18,134, VD = 104,935) were included. The mean difference in BMI between CS and VD was 0·72 kg·m^−2^ CI [0·27, 1·18], *p* = 0·002, I^2^ = 74% (Figure S3A). The OR for overweight was 1·28 CI [1·12, 1·47], *p* = 0·0004, I^2^ = 44% (Figure S3B). Eleven studies [13], [14], [39], [40], [42], [43], [45], [47], [50], [53], [60] (122,965 subjects; CS = 18,126, VD = 104,839) were suitable to investigate obesity (1·30 CI [1·05, 1·62], *p* = 0·02, I^2^ = 55% (Figure S3C).

Meta-regression showed the observed gender difference for BMI was not statistically significant (*p* = 0·25).

**Emergency/in-labour-CS:** Data from 4 studies [39], [43], [46], [53] (17,943 subjects; in-labour-CS = 281, VD = 17,662) were suitable for inclusion. There were no significant differences in BMI, overweight and obesity comparing in-labour-CS to VD (mean BMI difference: 0·48 kg·m^−2^ CI [−0·08, 1·04], *p* = 0·09, I^2^ = 7% (Figure S4A); overweight OR: 1·21 CI [0·95, 1·53], *p* = 0·12, I^2^ = 0% (Figure S4B); obesity OR: 1·26 CI [0·78, 2·05], *p* = 0·35, I^2^ = 39% (Figure S4C). The population available to investigate the incidence of obesity was 17,776 (in-labour-CS = 270; VD = 17,506), from 3 studies [39], [43], [53].

**Elective/pre-labour-CS:** Data from the same 4 studies [39], [43], [46], [53] (17,923 subjects; pre-labour-CS = 261, VD = 17,662) were included. There were no significant differences in BMI, overweight or obesity OR (mean BMI difference: 0·32 kg·m^−2^ CI [−0·21, 0·85], *p* = 0·24, I^2^ = 0% (Figure S5A); overweight OR 1·20 CI [0·93, 1·55], *p* = 0·15, I^2^ = 0% (Figure S5B); obesity OR: 1·13 CI [0·80, 1·59], *p* = 0·50, I^2^ = 0% (Figure S5C). The population for the evaluation of obesity was 17,758 (pre-labour-CS = 252; VD = 17,506) from 3 studies [39], [43], [53].

Subgroup analyses of CS against VD for all studies reporting type of CS showed similar effect-sizes to the overall analysis (mean BMI difference: 0·43 kg·m^−2^ [0·02, 0·85] (*p* = 0·04); overweight OR: 1·20 [1·01, 1.43] (*p* = 0·04); obesity OR: 1·21 [0·96; 1·54] *(p* = 0·11)). As there was no evidence of any difference in magnitude of effect when comparing type of CS (pre-labour-CS or in-labour-CS, compared to VD) on any outcome (BMI, overweight or obesity), a test comparing whether the effect varied between types of CS was not carried out.

Data from Cnattingius *et al* [47] (n = 103,941; not included in the sub-group analysis as the study only included women), showed significant mean BMI differences when comparing each type of CS to VD; in-labour-CS vs. VD: 1·40 kg·m^−2^ [1·28, 1·52] (*p*<0·00001), pre-labour-CS vs. VD: 1·00 kg·m^−2^ [0·89, 1·11] (*p<*0·00001). Interaction tests [63] show these differences in mean BMI difference between in-labour-CS and pre-labour-CS (compared to VD) to be statistically significant (*p*<0·001).

However, data from Svensson *et al* [51] (data on type of CS were only available for two years but were compared to the entire VD population; CS n = 533, VD n = 18,913; not included in the sub-group analysis as the study included only men), showed no significant mean BMI differences when comparing each type of CS to VD; in-labour-CS vs. VD: 0.30 kg·m^−2^ [−0.18, 0.78] p = 0.22, pre-labour-CS vs. VD: 0.20 kg·m^−2^ [−0.43,0.83] (*p = *0.54).

### Sensitivity analyses

Twenty-five percent of participants in Mi *et al* [42] and 40% of ACONF [39] were siblings but the statistical analysis did not reflect this. Furthermore, the implausible BMI values we noted in 1958 British Birth Cohort [53] brings into question the integrity of the dataset. To ensure that these potentially erroneous data did not bias the results, a sensitivity analysis was undertaken, excluding each study individually. Exclusion of these studies showed similar effect-sizes in all analyses (Table S1).

**Study quality:** Newcastle-Ottawa Scale scores (out of 7*) ranged from 3* to 7* (median = 4*; table 1). A sensitivity analysis of high quality studies (>4*) [13], [14], [40], [46], [53] showed greater effect-sizes (mean BMI difference: 0·69 kg·m^−2^ [0·36, 1·02], *p*<0.0001; overweight OR: 1·33 [1·20, 1·47], *p*<0·00001; obesity OR: 1.27 [1.03, 1.57], *p* = 0.02).

### Age of offspring

Mean offspring age at BMI measurement ranged from 18 to 69·6 years in the primary pooled gender analysis. Meta-regression showed a borderline significant decrease in mean difference in BMI with increasing mean offspring age, 0·023 kg·m^−2^ ([0·0008, 0·046], *p* = 0·06) less in mean BMI difference, per year increase in mean age (Figure S6).

### Funnel plots and publication bias

Visually the funnel plots (Figure S7, S8, S9) suggested asymmetry, although the Egger's test *p*-values were not statistically significant (*p* = 0·77; *p* = 0·42; *p* = 0·69 respectively).

A summary of all meta-analysis results is presented in table 2.

**Table 2:** Summary of all meta-analysis results.

	Number of subjects		Random effects		Heterogeneity			Fixed effects	
Comparison	CS	VD	Pooled result [95% CI]	p-value	I^2^	p-value	95% Prediction interval	Pooled result (95% CI)	p-value
**Mean difference BMI, all CS**	2995	34803	0·44 [0·17, 0·72]	0·002	39%	0.08	[−0·27, 1·15]	0·48 [0·30, 0·66]	0·00001
**OR overweight, all CS**	2995	34803	1·26 [1·16, 1·38]	0·00001	0%	0·92	[1.14, 1.40]	NA	NA
**OR obesity, all CS**	2975	34647	1·22[1·05, 1·42]	0.01	22%	0.24	[0.92, 1.92]	1.21 [1.07, 1.36]	0·002
**Mean difference BMI, all CS, males**	3791	36438	0·42 [0·18, 0·67]	0·0008	40%	0.07	[−0.19, 1.03]	0.49 [0.34, 0.63]	0.00001
**OR overweight, all CS, males**	3791	36438	1·21 [1·02, 1·43]	0·03	56%	0.0007	[0.75, 1.95]	NA	NA
**OR obesity, all CS, males**	3776	36378	1·33 [1·16, 1·53]	0·0001	8%	0.36	[1.01, 1.75]	1.34 [1.19, 1.50]	0.00001
**Mean difference BMI, all CS, females**	18134	104935	0·72 [0·27, 1·18]	0·002	74%	0·00001	[−0.66, 2.10]	NA	NA
**OR overweight, all CS, females**	18134	104935	1·28 [1·12, 1·47]	0·0004	44%	0.05	[0.90, 1.82]	NA	NA
**OR obesity, all CS, females**	18126	104839	1·30 [1·05, 1·62]	0·02	66%	0·01	[0.74, 1.19]	NA	NA
**Mean difference BMI, in-labour-CS**	281	17662	0·48 [−0·08, 1·04]	0·09	7%	0·36	[−0.89, 1.85]	0.49 [−0.04, 1.03]	0.07
**OR overweight, in-labour-CS**	281	17662	1·21[0·95, 1·53]	0·12	0%	0·72	[0.72,2.03]	NA	NA
**OR obesity, in-labour-CS**	270	17506	1·26 [0·78, 2·05]	0·35	39%	0·20	[0.01, 127]	1.29 [0.93, 1.78]	0.12
**Mean difference BMI, pre-labour-CS**	261	17662	0·32 [−0·21, 0·85]	0·24	0%	0·47	[−0.84, 1.48]	NA	NA
**OR overweight, pre-labour-CS**	261	17662	1·20 [0·93, 1·55]	0·15	0%	0·85	[0.68, 2.10]	NA	NA
**OR obesity, pre-labour-CS**	252	17506	1·13 [0·80, 1·59]	0·50	0%	0·82	[0.12, 10.3]	NA	NA
**Mean difference BMI, all CS, studies with CS type data only**	542	17662	0·43 [0·02, 0·85]	0·04	10%	0.34	[−0.67,1.53]	0.44 [0.05, 0.82]	0.03
**OR overweight, all CS, studies with CS type data only**	542	17662	1·20 [1·01, 1.43]	0·04	0%	0.98	[0.82,1.76]	NA	NA
**OR obesity, all CS, studies with CS type data only**	522	17506	1·21 [0·96; 1·54]	0·11	0%	0.38	[0.25,5.78]	NA	NA
**Mean difference BMI, all CS, high quality studies only**	2195	16817	0·69 [0·36, 1·02]	0.0001	36%	0.18	[−0.20,1.58]	0.64 [0.42, 0.86]	0.00001
**OR overweight, all CS, high quality studies only**	2195	16817	1·33 [1·20, 1·47]	0·00001	0%	0.91	[1.13,1.56]	NA	NA
**OR obesity, all CS, high quality studies only**	2175	16661	1.27 [1.03, 1.57]	0.02	39%	0.18	[0.79,2.02]	1.27 [1.09, 1.48]	0.002

## Discussion

In this large systematic review and meta-analysis investigating the association between mode of delivery and adult BMI, overweight and obesity, we found an average increase in BMI of almost 0·5 kg·m^−2^ in subjects delivered by CS compared to VD, and an increased odds of overweight and obesity >20%; these findings are consistent across sexes. We found some indication that studies of younger populations showed greater effect-size.

Given that rate of CS has changed over time; driven partially by changing clinical practice and by the rise of the maternal choice CS, it is possible that the impact of CS on outcomes may be different dependent on the birth year of the cohort. Studies with a higher background CS rate, and studies where participants were born after 1975, have higher effect sizes and narrower confidence intervals (figure S10, S11). The narrower confidence intervals may be in part a reflection of the increased size of the CS groups. These figures demonstrate that inclusion of the older cohorts in our meta-analysis is likely to attenuate the effect sizes we report. This emphasises that the relationship between mode of delivery and later life outcomes is likely to be of increasing importance in future populations.

There are strengths and limitations to our systematic review and meta-analysis. A key strength is the large population of 142,702 subjects, from ten countries, spanning four continents; a large part of which have not been previously published in this form. Pre-registration of an *a priori* protocol prevented *post hoc* modifications to analyses, decreasing the risk of bias. Furthermore, heterogeneity was low in all primary analyses given all studies were observational. Prediction intervals were calculated to indicate the likely range of study effects in the presence of heterogeneity. We showed that while on average BMI was higher in the CS group, this may not occur in all settings as the prediction interval ranged from −0.27 to 1.15 kg·m^−2^. A similar finding was observed for obesity, but there was minimal heterogeneity in the analysis of overweight. All studies were of comparable high quality, limited only by low follow-up rates (mean follow-up rate across all included studies was 60%) and exclusion of studies with lower quality scores increased effect-sizes.

Despite these strengths, there were limitations. Funnel plot asymmetry suggested the possibility of reporting bias, but Egger's Test was statistically non-significant and as the outlying studies are in the opposite direction to the pooled results, any bias present probably attenuates rather than accentuates the findings. There was no evidence of a statistically significant gender difference in effect. Furthermore, gender-specific analyses showed higher levels of heterogeneity than the main results, possibly because the population size for each study is halved so the study estimates are less precise.

Collection of mode of delivery data in cohort studies was poor; these data were recorded in only 16 studies out of 33 identified (<15% of the total population of potential studies identified). Also, considerable aggregate data were unavailable for inclusion: 16 studies (a combined population >260,000 subjects) were identified in which relevant data had been collected, yet these were only available from 13, (142,280 subjects). A lack of patient level data prevented adjustment for confounders.

Findings from our meta-analysis are consistent with findings from other investigators. Shortly after completing our review, Li *et al* [64] reported 50% higher odds of obesity in adults born by CS compared to their VD counterparts from a systematic review and meta-analysis. In contrast to our findings: a greater effect size among higher quality studies, Li *et al* [64] found the association between CS and overweight/obesity no longer significant in a subgroup analysis limited to high quality studies (including 2 studies and 6,354 subjects). The small size of the Li *et al* subgroup analysis limits its findings, and we suggest that the apparently conflicting results of our subgroup analysis limited to high quality studies reflects the more inclusive nature of our review. Unlike Li *et al* [64], we were not limited by a small dataset and were therefore able to perform subgroup analyses, investigating the effects of gender on overall outcome, which they had initially set out to do. Furthermore, the availability of more data, in addition to contacting the authors for further information, we were able to present the risk of overweight and obesity separately, as well as specific BMI differences. Li *et al* [64] were however, able to present an adjusted pooled analysis, which due to a lack of patient level information we were unable to do. However, of note is that the adjusted estimate correlates closely with the unadjusted estimates we have shown through this study.

Goldani *et al* [13] reported the odds of obesity in adults born by CS to be 50% higher, and importantly, when they adjusted for confounders (see table 1), the effect of CS on offspring BMI was slightly increased. Barros *et al* [14] found no effect of CS on incidence of adult offspring obesity either before, or after, adjustment for a range of confounders (see table 1). This may be due to lack of power in the Barros study [14], as suggested by Li *et al* [65]. One key difference between the two Brazilian studies is that Barros *et al* [14] were able to adjust the data for maternal pre-pregnancy height and weight unlike Goldani *et al.* This could account for the differences seen, but the unadjusted results from the Barros study still showed no significant difference in obesity between the two groups.

A key consideration is whether the associations we have identified between mode of delivery and offspring outcome are causal or reflect confounding. Plausible causal factors that might lead from CS to greater risk of obesity include differences in offspring microbiome as this differs between CS and VD neonates [66], leading to increased energy harvesting [67], and a lower rate of breastfeeding [68], in turn associated with greater risk of later-life overweight and obesity [69]. Non-exposure to labour also results in persistence of fetal gene expression and altered metabolism [9]. Nonetheless several factors associated with increased risk of CS are also associated with increased BMI in offspring, including high maternal BMI [70], gestational diabetes [71], and lower socioeconomic status [72], [73]. Of these, maternal obesity is probably the most significant confounding factor in the relationship between CS and offspring BMI. This needs elucidation in datasets which can be properly controlled for maternal BMI.

To our best knowledge there have been no previous attempts to evaluate the effect of exposure to labour on later-life BMI, by examining outcomes in in-labour-CS and pre-labour-CS. When data were stratified by type of CS, we found no significant differences in adult BMI, overweight and obesity. However, these data were provided in only 5 studies, and the number of CS-delivered participants included in these subgroup analyses was <10% of the total number of CS deliveries in the main meta-analysis. Given the lack of power we do not feel that reliable conclusions can be drawn regarding the possibility of an impact of exposure to labour on study outcomes.

Our meta-analysis shows reduced association between CS and later-life BMI in older subjects. Exposure to post-natal obesogenic environmental factors increases with age, and may mask the association between CS and increased BMI in later life. However, as overweight and obesity track across time and are amplified in an obesogenic environment it seems unlikely that any impact of CS is truly attenuated with age. Hence we consider this is most likely due to the rising prevalence of obesity driving a larger effect-size in younger populations, although, longitudinal follow-up of these cohorts will be necessary to establish this with certainty.

In 2010 the WHO identified increased short-term adverse effects to mother and baby [74] following CS without medical indication. Controversially despite this evidence, and without considering possible long-term adverse outcomes, the highly influential UK NICE guidelines [75] were modified in 2011 to support offering CS to women who, after discussion with a mental health expert, feel that VD is an unacceptable option. Concern has been expressed that this guideline will further serve to fuel the already high and rapidly rising CS delivery rate worldwide. Associations between CS and increased later life risk of overweight and obesity are of significant relevance to population health. This requires that the possibility of a causal relationship be addressed as a matter of urgency, at the very least to provide the sound evidence required for women to make an informed decision regarding the advisability of delivery by CS in the absence of medical indication.

## Supporting Information

Figure S1 (TIF)

Figure S2 (TIF)

Figure S3 (TIF)

Figure S4 (TIF)

Figure S5 (TIF)

Figure S6 (TIF)

Figure S7 (TIF)

Figure S8 (TIF)

Figure S9 (TIF)

Figure S10 (TIF)

Figure S11 (TIF)

Table S1 (TIF)

Checklist S1 (DOC)

Protocol S1 (PDF)
