# Correction: Mode of Delivery and Offspring Body Mass Index, Overweight and Obesity in Adult Life: A Systematic Review and Meta-Analysis

**DOI:** 10.1371/journal.pone.0097827

**Published:** 2014-05-13

**Authors:** The PLOS ONE Staff

The number of participants for this study is incorrectly listed in several locations in the published manuscript:

In the Abstract and Results sections, the number of participants should be “163, 796” rather than “163, 753.”

In the Discussion section, the number of participants should be “163, 796” rather than “142, 702.”

The number of studies and the number of participants are incorrectly cited in the 5^th^ paragraph of the discussion. The correct paragraph is:

“Collection of mode of delivery data in cohort studies was poor; these data were recorded in only 18 studies out of 35 identified (<15% of the total population of potential studies identified). Also, considerable aggregate data were unavailable for inclusion: 18 studies (a combined population >260,000 subjects) were identified in which relevant data had been collected, yet these were only available from 15, (163,796 subjects). A lack of patient level data prevented adjustment for confounders.”

## References

[1] Darmasseelane K, Hyde MJ, Santhakumaran S, Gale C, Modi N (2014) Mode of Delivery and Offspring Body Mass Index, Overweight and Obesity in Adult Life: A Systematic Review and Meta-Analysis. PLoS ONE 9(2): e87896 doi:10.1371/journal.pone.0087896 24586295 10.1371/journal.pone.0087896 PMC3935836

