# Supplementary figures and images for: Mode of Delivery and Offspring Body Mass Index, Overweight and Obesity in Adult Life: A Systematic Review and Meta-Analysis
Source: PLoS One. 2014 Feb 26;9(2):e87896. doi: 10.1371/journal.pone.0087896 (PMC3935836; doi:10.1371/journal.pone.0087896)

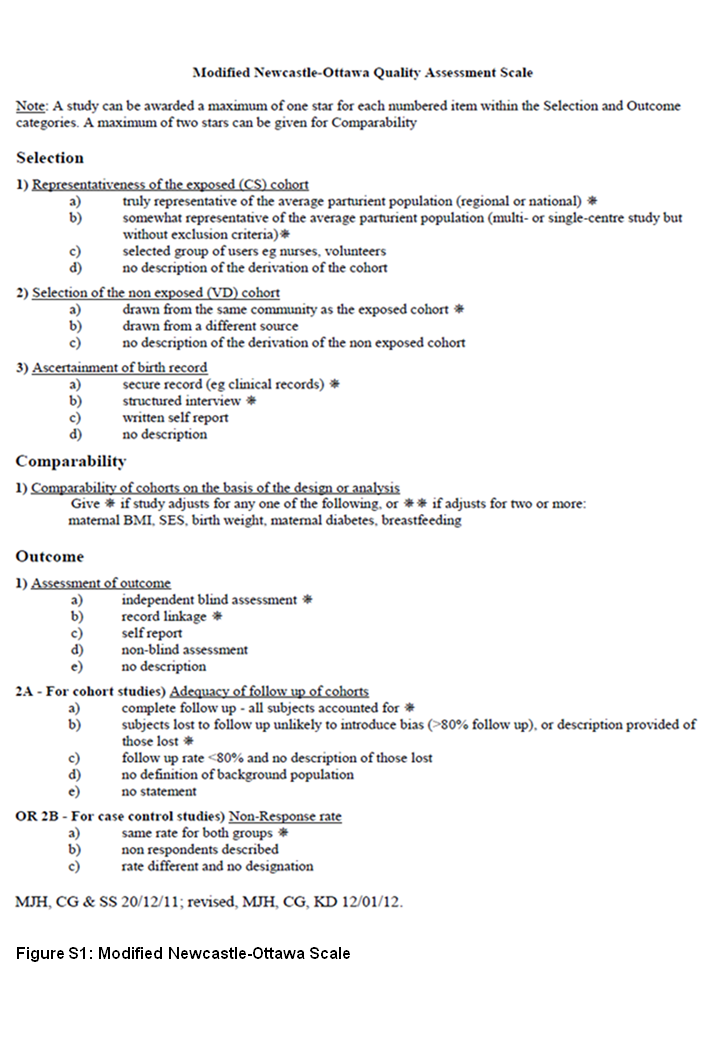

Supplement: Figure S1 — (TIF) [file pone.0087896.s001.tif]

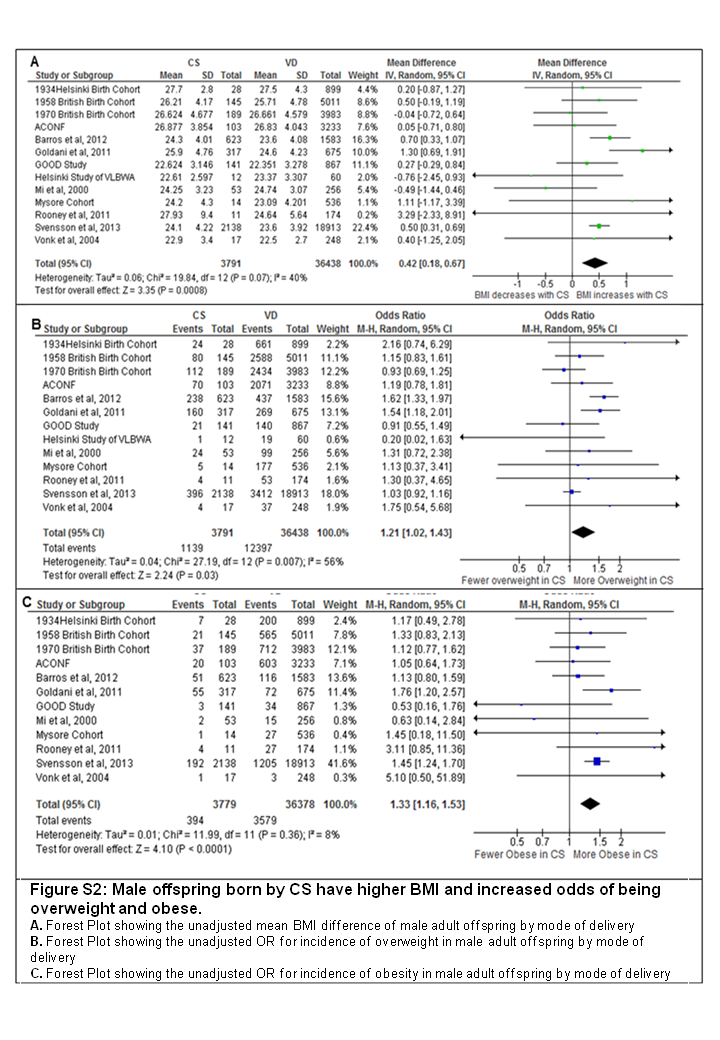

Supplement: Figure S2 — (TIF) [file pone.0087896.s002.tif]

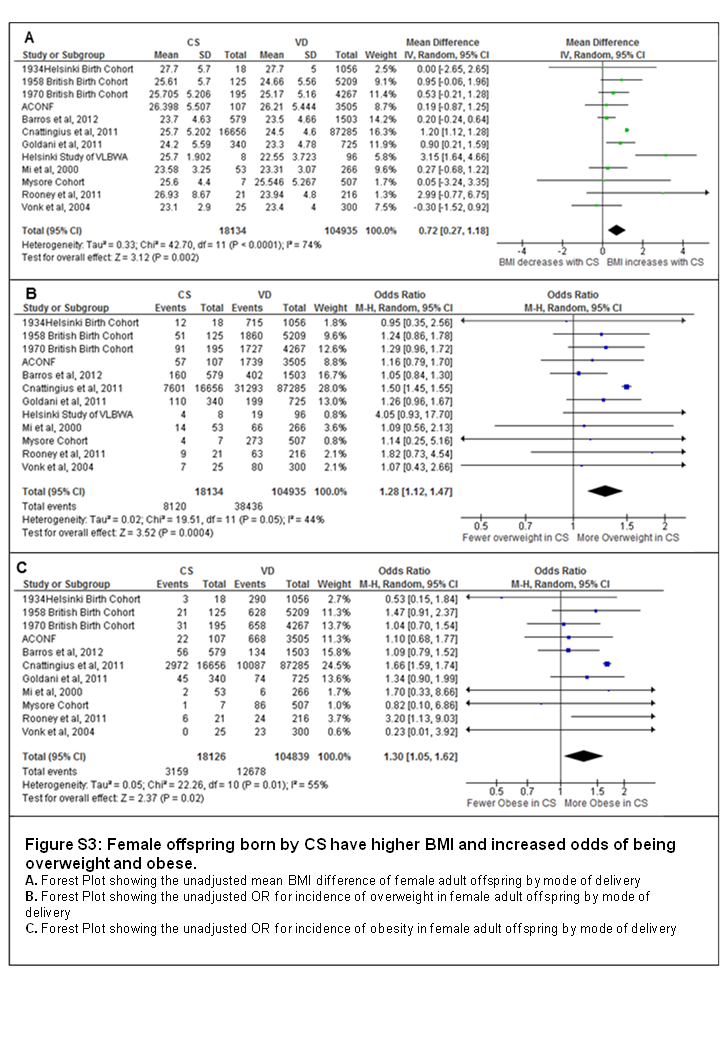

Supplement: Figure S3 — (TIF) [file pone.0087896.s003.tif]

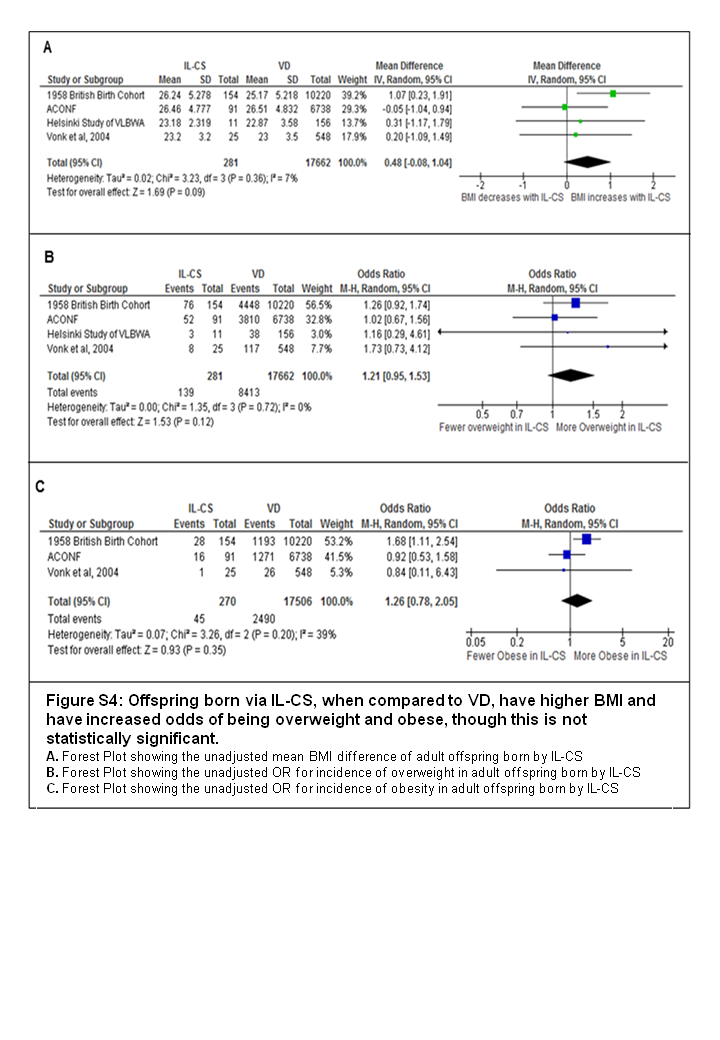

Supplement: Figure S4 — (TIF) [file pone.0087896.s004.tif]

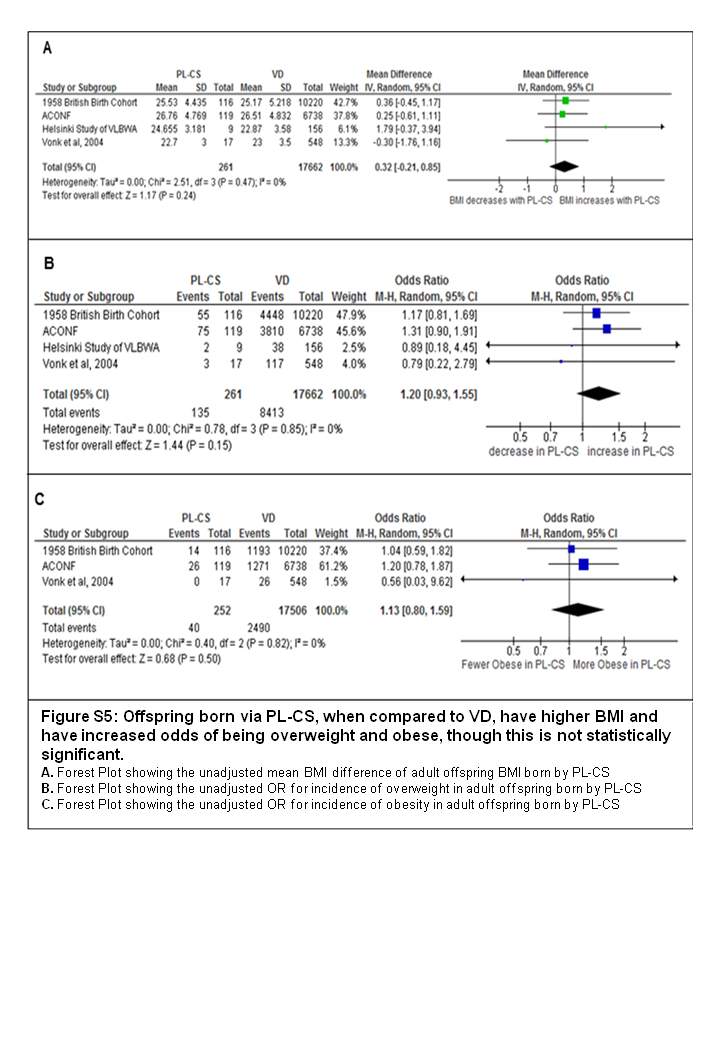

Supplement: Figure S5 — (TIF) [file pone.0087896.s005.tif]

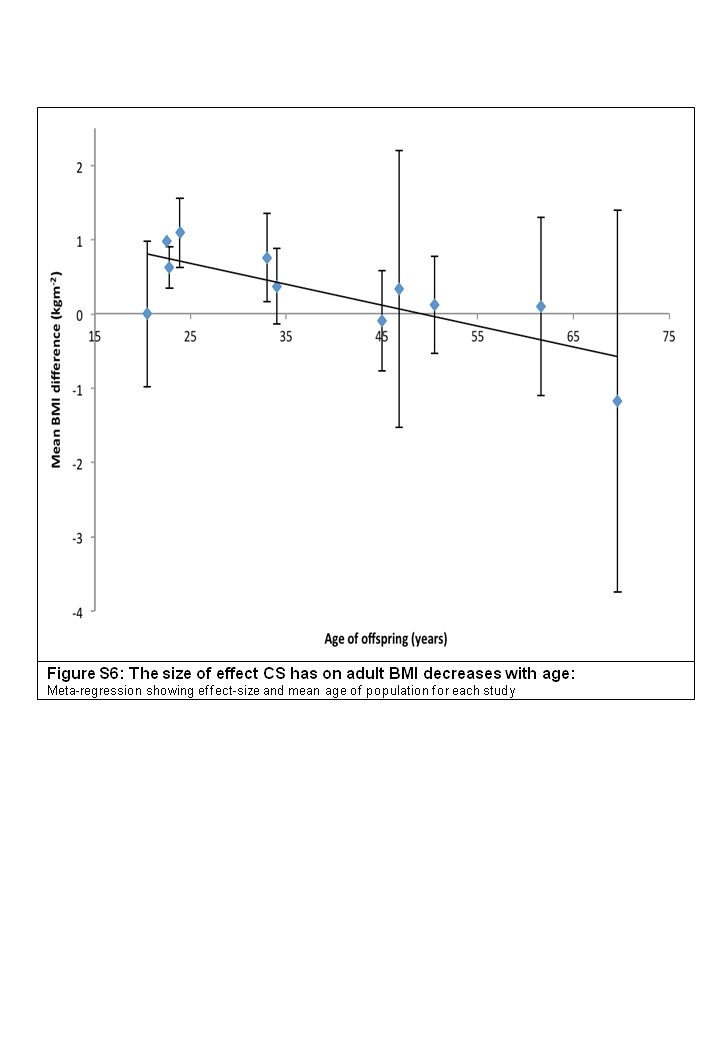

Supplement: Figure S6 — (TIF) [file pone.0087896.s006.tif]

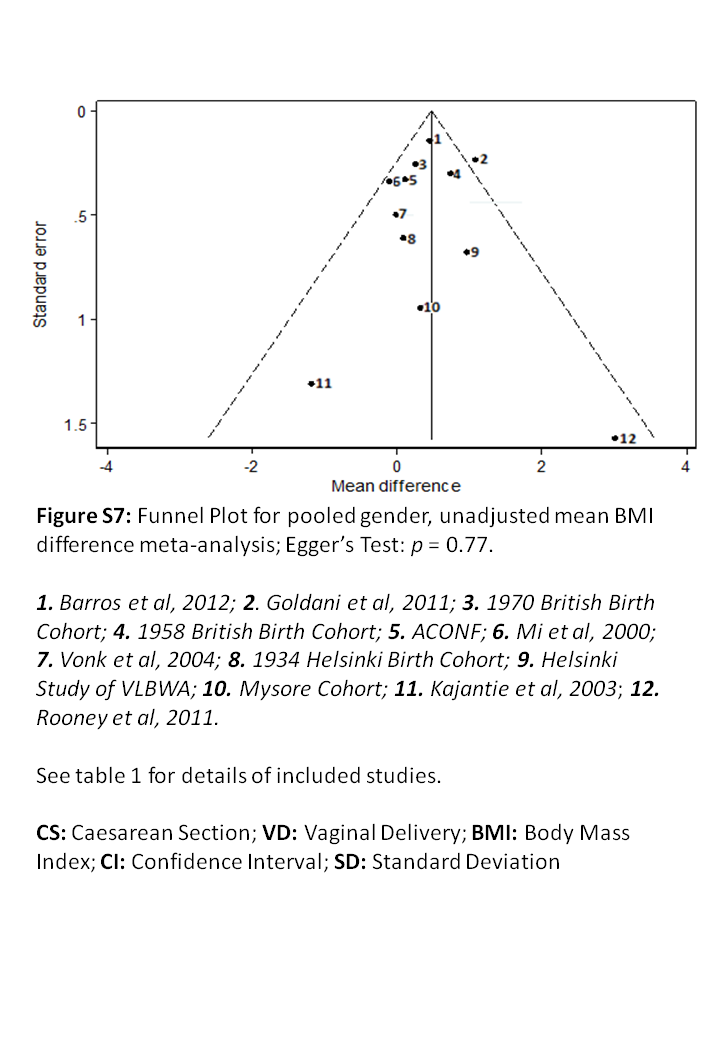

Supplement: Figure S7 — (TIF) [file pone.0087896.s007.tif]

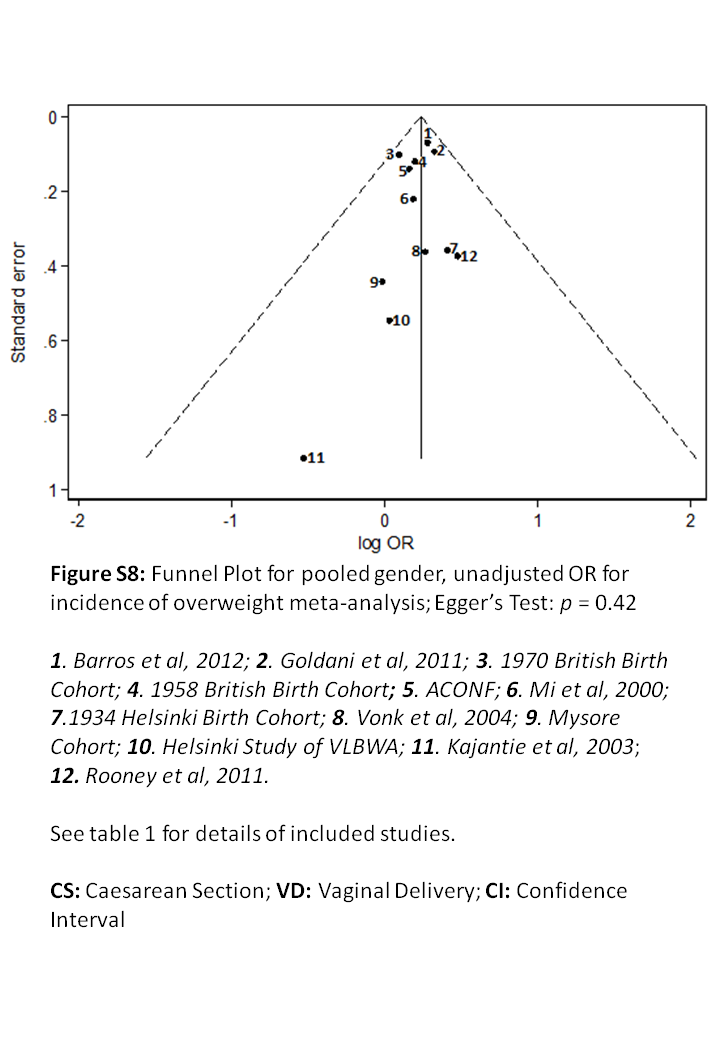

Supplement: Figure S8 — (TIF) [file pone.0087896.s008.tif]

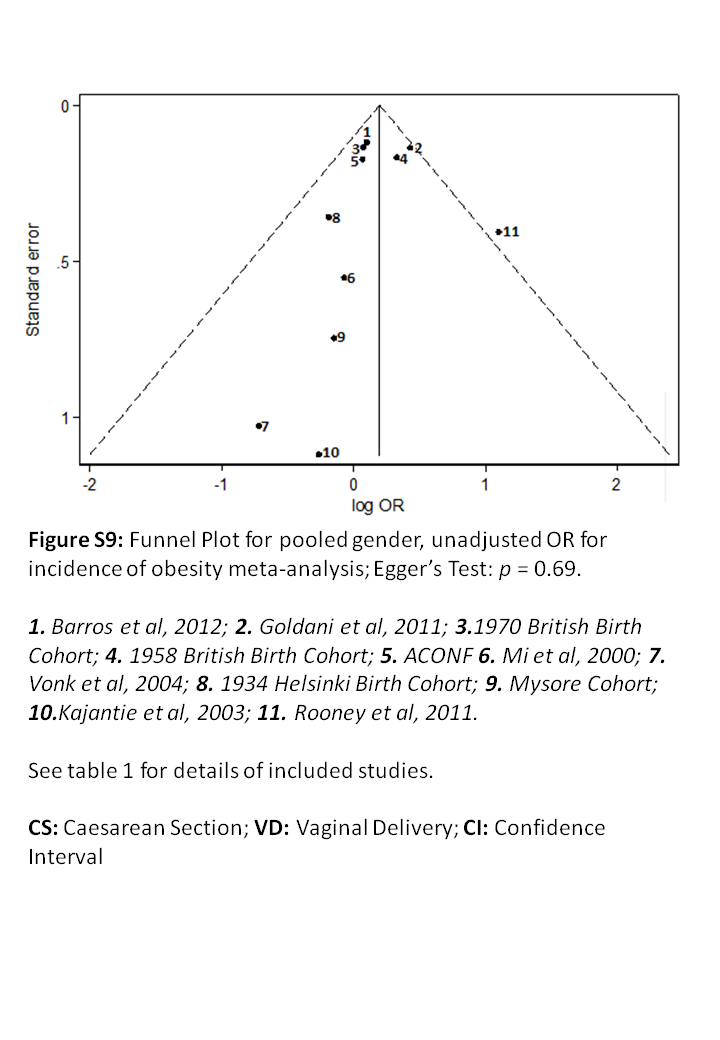

Supplement: Figure S9 — (TIF) [file pone.0087896.s009.tif]

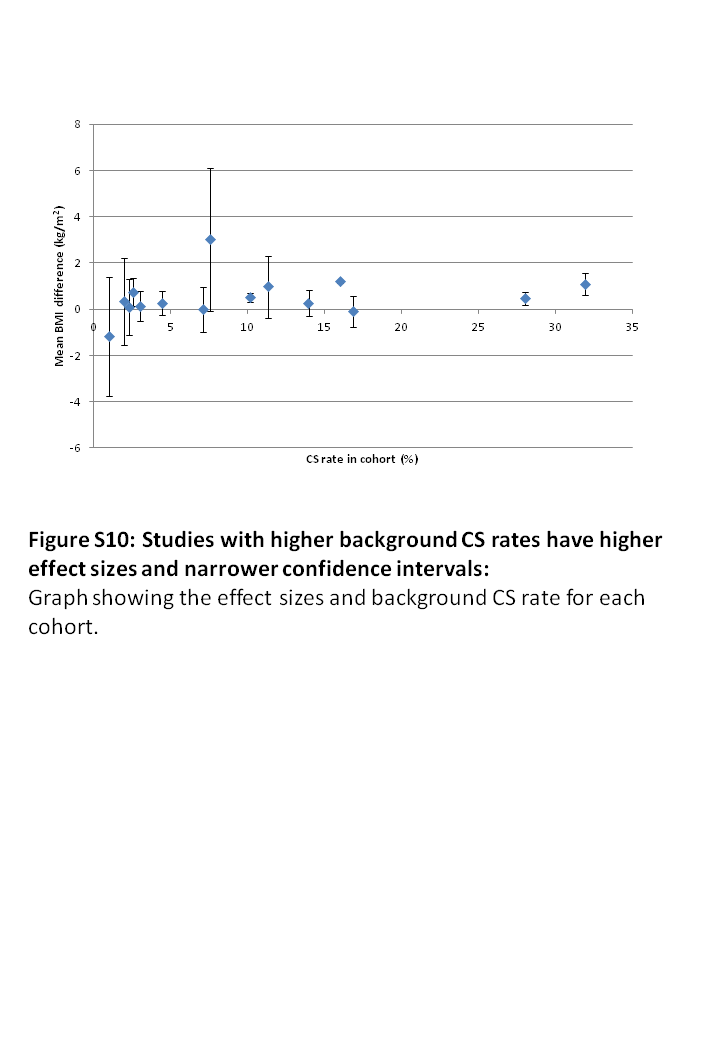

Supplement: Figure S10 — (TIF) [file pone.0087896.s010.tif]

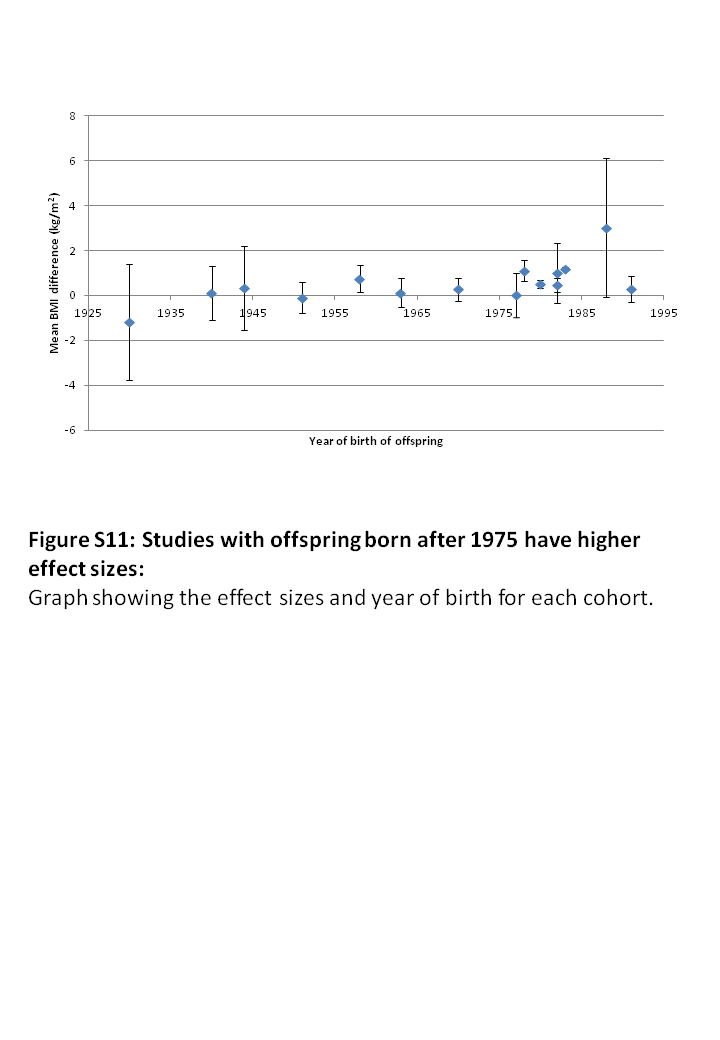

Supplement: Figure S11 — (TIF) [file pone.0087896.s011.tif]
