# Supplementary material for: Mode of Delivery and Offspring Body Mass Index, Overweight and Obesity in Adult Life: A Systematic Review and Meta-Analysis
Source: PLoS One. 2014 Feb 26;9(2):e87896. doi: 10.1371/journal.pone.0087896 (PMC3935836; doi:10.1371/journal.pone.0087896)
Supplement: Protocol S1 — (PDF) [file pone.0087896.s014.pdf]

PROSPERO
International prospective register of systematic reviews

NHS
National Institute for Health Research

Mode of delivery and offspring Body Mass Index, overweight and obesity in adult life: a systematic review and meta-analysis

Matthew Hyde, Neena Modi, Christopher Gale, Karthik Darmasseelane, Shalini Santhakumaran

Citation
Matthew Hyde, Neena Modi, Christopher Gale, Karthik Darmasseelane, Shalini Santhakumaran . Mode of delivery and offspring Body Mass Index, overweight and obesity in adult life: a systematic review and meta-analysis. PROSPERO 2011:CRD42011001851 Available fromhttp://www.crd.york.ac.uk/PROSPERO/display_record.asp?ID=CRD42011001851

Review question(s)
Is delivery by Caesarean section, when compared to vaginal delivery, associated with higher offspring Body Mass Index in adult life?
Is delivery by Caesarean section, when compared to vaginal delivery, associated with higher risk of offspring overweight or obesity in adult life?

Searches
[1] A search will be performed using the PubMED database to identify initial studies using the following search strategy: (Parturition OR Delivery, Obstetric OR Cesarean) AND (obesity OR Body Mass Index OR Overweight), limited to human studies. No language or publication period restriction will be applied.
Abstracts for these studies will be obtained for assessment and inclusion will be determined using the criteria outlined in Section 22 (Types of study to be included initially).
[2] A search of Google Scholar and Web of Science will be undertaken, using the following search (caesarean OR cesarean OR caesarian OR cesarian) AND (obesity OR Body Mass Index OR Overweight) AND (Adult) AND (Offspring).
Abstracts for these studies will be obtained for assessment and inclusion will be determined using the criteria outlined in Section 22 (Types of study to be included initially).
[3] A manual search of all appropriate references (identified as relevant from their citation within the article) cited in included studies and identified review articles will be performed.
Abstracts for these studies will be obtained for assessment and inclusion will be determined using the criteria outlined in Section 22 (Types of study to be included initially).

Types of study to be included
There are no restrictions on the types of study design eligible for inclusion. All study types reporting Body Mass Index/obesity/overweight in adult life, categorised by method of delivery, will be included.

Condition or domain being studied
Mode of delivery has been implicated as an event that might influence health and the risk of disease in adult life (Hyde MJ et al (2011) Biological Reviews, DOI: 10.1111/j.1469-185X.2011.00195.x).
A few published reports support the hypothesis that the risk of overweight/obesity is affected (eg Goldani HS et al, Am J Clin Nutr 2011; 93:1344)
Delivery by Caesarean section and pre-labour Caesarean section is growing, as is obesity and overweight. Individual studies suggest an association between Caesarean section and the risk of obesity. There are plausible candidate mechanisms that might underpin such as association. We therefore propose to conduct a systematic review and meta-analysis of available data on adult Body Mass Index and weight in relation to mode of delivery.

Participants/ population
Outcomes are evaluated in adults (i.e. age 18 or above).

Intervention(s), exposure(s)
Caesarean section (Pre-labour or in-labour)

Comparator(s)/ control
Vaginal delivery

Context
The review will review all identified studies

Outcome(s)

Primary outcomes
Difference in Body Mass Index (kg/m^2) measured between control and intervention group in adult life (18years or older)
Difference in prevalence of overweight (Body Mass Index greater than 25kg/m^2) /obese (Body Mass Index greater than 30kg/m^2) measured between control and intervention group in adult life (18years or older)

Secondary outcomes
None

Data extraction, (selection and coding)
Titles and abstracts (where available), of studies identified from the initial searches will be independently assessed by two reviewers for possible inclusion [KD and MJH]. The full text of all potentially eligible studies that have been identified will then be appraised by two assessors independently [KD and CG]. Where there is disagreement over eligibility for inclusion, this will be referred to a meeting of all authors. Data will be extracted independently onto a pre-piloted data collection form [KD and CG]. Quality of the included studies will be assessed using standard criteria, looking for potential bias [All authors independently].
The following data will be extracted for each study: type of study (cross sectional/longitudinal; retrospective/prospective); country of study; study population, setting and demographics of research subjects (offspring: gender, age at study, maternal Body Mass Index during pregnancy, maternal diabetes during pregnancy); details of intervention (type of delivery, year/period of deliveries being studied); inclusion and exclusion criteria; recruitment and study completion rates; outcome measurements; and adjusted analyses

performed (yes/no and factors adjusted for).
Missing data will be requested from study authors by MJH.

Risk of bias (quality) assessment

[1] Review level Bias:
Bias from small study effects will be assessed visually using a funnel plot and Egger's test; where asymmetry is evident on the Funnel Plot a trim and fill analysis will be used. Possible causes for asymmetry other than publication bias (e.g. between-study heterogeneity) will also be considered. Where population based cohorts are identified by the search strategy but outcomes are not reported on the basis of mode of delivery, attempts will be made to extract this information for these studies by contacting the data custodian in order to reduce effects of publication bias.
[2] Study level bias:
A modified Newcastle-Ottawa scale will be used to assess methodological quality of each individual study (carried out independently by SS and KD, discrepancies to be resolved by group discussion). A subgroup analysis of the studies scoring 7 out of 7 stars will be conducted. Ranked forest plots (on the basis of the score out of 7 that each study achieves) will be produced for primary outcomes, to allow assessment of study quality on effect size.

Strategy for data synthesis

Data collected will be systematised into a table. All studies identified in the systematic review will be included in the table whether or not they are included in the meta-analysis. A narrative description of these studies will be produced.
Where studies are comparable on the basis of study population, intervention and outcome measure, the results will be pooled in a fixed-effects meta-analysis, with mean differences, 95% confidence intervals and two sided p-values. Heterogeneity will be assessed using the Chi-squared test for the Q statistic and calculation of I-squared, an estimate of the proportion of variance due to between-study heterogeneity. Where evidence of heterogeneity is present (p=value from Chi-squared test <0.05) a random effects meta-analysis will be used with meta-regression and subgroup analysis to explore sources of heterogeneity arising from study characteristics. Where between-study heterogeneity is very high (I-squared over 80%) and is not explained through differences in study characteristics we will re-consider whether quantitative data synthesis is appropriate.
Data from studies adjusting for confounders will be synthesised in a separate meta-analysis to the unadjusted data.

Analysis of subgroups or subsets

[1] Subgroup analysis: For each subgroup analysis we will use meta-regression to investigate whether the effects are significantly different in the subgroups. Subgroup comparisons to be carried out:
a) Type of Caesarean section (pre labour and in labour), and
b) Gender.
[2] Meta regression: To investigate heterogeneity for continuous variables, we will use scatter plots of effect size against study characteristic and meta-regression. Meta regression analyses to be carried out:
a) Age of offspring at assessment,
b) Study quality, and
c) Difference in Maternal Body Mass Index between Caesarean Section and Vaginal Delivery groups.
[3] Sensitivity analysis: Sensitivity analysis will be used to test whether conclusions are robust to alterations in the inclusion criteria. Sensitivity analyses to be carried out:
a) Exclusion of studies including preterms (<37 gestational weeks), and
b) Exclusion of studies including diabetic pregnancies

Dissemination plans

The results of this review will be presented at meetings of relevant societies and interest groups. It will be written up for peer-reviewed publication.

Contact details for further information

Matthew Hyde
Neonatal Medicine,
Imperial College London,
4th Floor, Lift Bank D,
Chelsea and Westminster Hospital,
369 Fulham Road,
LONDON,
SW10 9NH.
matthew.hyde02@imperial.ac.uk

Organisational affiliation of the review

Imperial College London
http://www1.imperial.ac.uk/departmentofmedicine/divisions/infectiousdiseases/paediatrics/neonatalmedicine/

Review team

Dr Matthew Hyde, Imperial College London
Professor Neena Modi, Imperial College London
Dr Christopher Gale, Imperial College London
Mr Karthik Darmasseelane, Imperial College London
Ms Shalini Santhakumaran , Imperial College London

Details of any existing review of the same topic by the same authors

None

Anticipated or actual start date

16 December 2011

Anticipated completion date

14 June 2012

Funding sources/sponsors

Funding source: Imperial College project funding

Conflicts of interest

None known

Other registration details

Imperial College London

Language

English

Country

England

Subject index terms status

Subject indexing assigned by CRD

Subject index terms

Adult; Body Mass Index; Cesarean Section; Delivery, Obstetric; Humans; Obesity; Overweight; Risk Factors;

Reference and/or URL for protocol

http://www.crd.york.ac.uk/PROSPEROFILES/1851_PROTOCOL_20111120.pdf

Date of registration in PROSPERO

23 December 2011

Date of publication of this revision

23 December 2011

**Stage of review at time of this submission**	**Started**	**Completed**
Preliminary searches	Yes	No
Piloting of the study selection process	No	No
Formal screening of search results against eligibility criteria	No	No
Data extraction	No	No
Risk of bias (quality) assessment	No	No
Data analysis	No	No
Prospective meta-analysis	No	No

PROSPERO
This information has been provided by the named contact for this review. CRD has accepted this information in good faith and registered the review in PROSPERO. CRD bears no responsibility or liability for the content of this registration record, any associated files or external websites.
